# The Crosstalk Mechanism of EGFR and ER in EGFR-Mutant Lung Adenocarcinoma

**DOI:** 10.3390/cells15020098

**Published:** 2026-01-06

**Authors:** Ying-Yi Chen, Wei-Ting Huang, Yu-Fu Su, Yi-Jen Hung, Hao-Ai Shui, Yi-Shing Shieh, Tsai-Wang Huang

**Affiliations:** 1 Graduate Institute of Medical Sciences, College of Medicine, National Defense Medical University, Taipei 114202, Taiwan; addgujfjui@gmail.com (Y.-Y.C.); or haoai@office365.ndmctsgh.edu.tw (H.-A.S.); ndmcyss@mail.ndmctsgh.edu.tw (Y.-S.S.); 2 Division of Thoracic Surgery, Department of Surgery, Tri-Service General Hospital, National Defense Medical University, Taipei 114202, Taiwan; 3 Department of Dentistry, Tri-Service General Hospital, National Defense Medical University, Taipei 114202, Taiwan; 4 Department of Radiation Oncology, Tri-Service General Hospital, National Defense Medical University, Taipei 114202, Taiwan; 5 Division of Endocrinology and Metabolism, Tri-Service General Hospital, National Defense Medical University, Taipei 114202, Taiwan

**Keywords:** lung adenocarcinoma, epidermal growth factor receptor, estrogen receptor, breast cancer

## Abstract

Breast cancer and lung adenocarcinoma share common features, including female predominance and the expression of estrogen receptor (ER) and epidermal growth factor receptor (EGFR) during carcinogenesis. Patients with breast cancer have a significantly higher risk of developing second primary lung cancer than those without breast cancer. ER beta expression is associated with resistance to EGFR tyrosine kinase inhibitors (TKIs) in EGFR-mutant lung adenocarcinoma, indicating a potentially important interaction between ER and EGFR. However, the mechanisms underlying this crosstalk remain poorly understood. Our clinical data showed a significant correlation between antiestrogen treatment for breast cancer and mutant EGFR expression (*p* = 0.021) in lung adenocarcinoma patients. In vitro, tamoxifen upregulated phosphorylated EGFR (p-EGFR) in EGFR-mutant lung adenocarcinoma cell lines. Heparin-binding EGF-like growth factor was identified as a key mediator from the ER pathway that stimulates p-EGFR. Tamoxifen counteracts estrogen’s effect and restores p-EGFR upregulation. Furthermore, coadministration of tamoxifen and the EGFR TKI gefitinib potentially inhibited p-EGFR expression in EGFR-mutant lung adenocarcinoma. Regular follow-up with chest computed tomography is recommended for patients with breast cancer. For those diagnosed with both ER-positive breast cancer and EGFR-mutant lung adenocarcinoma, combined tamoxifen and EGFR TKI therapy may offer an effective targeted treatment strategy.

## 1. Introduction

Lung cancer and breast cancer, despite advancements in diagnosis and treatment, continue to be leading causes of death in Taiwan, with mortality rates increasing annually [1]. Both cancers share common features, such as female predominance, estrogen receptor (ER) expression, and epidermal growth factor receptor (EGFR) expression, during carcinogenesis [2,3,4,5,6]. Recent studies have highlighted the close relationship between ER expression and EGFR in patients with lung adenocarcinoma who had a history of breast cancer [7,8,9,10]. This has led to an increased focus on understanding the role of ER in EGFR-mutant lung adenocarcinoma. Despite contradictory findings [11,12,13,14], the potential interaction between ER and EGFR in lung adenocarcinoma indicates a complex crosstalk mechanism that could significantly influence treatment. The conflicting results from literature review about the role of estrogen in lung cancer may be attributed to the effects of lung cancer subtypes. Therefore, we focused on the role of ER in patients with EGFR-mutant lung adenocarcinoma. However, the mechanisms of this crosstalk remain poorly understood, underscoring the need for further research to clarify the role of ER in EGFR-mutant lung adenocarcinoma. The findings could lead to the development of more effective therapeutic approaches, ultimately improving patient outcomes. Therefore, this research from bench to clinical study utilized our database of patients with lung adenocarcinoma and performed basic experiments to prove the close relationship and key prognostic factors between EGFR-mutant lung adenocarcinoma and breast cancer.

## 2. Materials and Methods

### 2.1. Reagents

The following reagents were used: tamoxifen (Sigma #T5648, St. Louis, MO, USA), β-Estradiol (Sigma E8875, St. Louis, MO, USA), fulvestrant (Sigma #I4409, St. Louis, MO, USA), Heparin-binding epidermal growth factor–like growth factor (Hb-EGF) antibody (R&D System #AF-259-SP, Minneapolis, MN, USA), transforming growth factor (TGF) alpha monoclonal antibody (MF9) (#MA5-13907, Waltham, MA, USA), SRC polyclonal antibody (#44-656G, Waltham, MA, USA), osteopontin monoclonal antibody (7C5H12) (#MA5-17180, Waltham, MA, USA), anti-EGFR monoclonal antibody (# MA5-13319, Waltham, MA, USA), phospho-EGFR (Tyr1173) polyclonal antibody (# 44-794G, Waltham, MA, USA), and ER alpha monoclonal antibody (SP1) (#MA5-14501, Waltham, MA, USA), phospho-Extracellular Signal-Regulated Kinase (p-ERK) (CST #4695, Danvers, MA, USA), phospho- Protein Kinase B (p-AKT S473) (CST #4060, Danvers, MA, USA).

### 2.2. Cell Lines and Culture Conditions

The non-small cell lung cancer (NSCLC) lines PC9 (ECACC 90071810) and HCC827 (ATCC CRL-2868) were used as models harboring EGFR exon 19 deletion mutations. The Taiwanese lung adenocarcinoma cell lines CL1-0 (Cellosaurus CVCL_3871) and its highly invasive derivative CL1-5 (Cellosaurus CVCL_D521) were included for invasion and metastasis studies. Additionally, the HER2-overexpressing breast cancer line AU565 (ATCC CRL-2351) was utilized for comparative analysis. These cancer cell lines were cultured in RPMI 1640 medium.

For drug resistance assays, Gefitinib-resistant (GR) derivatives were obtained commercially: PC9-GR (Cellosaurus CVCL_E7RA) was purchased from Sigma-Aldrich, and HCC827-GR (Cellosaurus CVCL_S705) was acquired from Merck/MilliporeSigma. Normal lung controls included BEAS-2B (ATCC CRL-9609), an immortalized normal bronchial epithelial line transformed via Ad12-SV40 hybrid virus, and HPAEpiC (ScienCell Catalog No. 3200), a primary human pulmonary alveolar epithelial cell line obtained from ScienCell Research Laboratories. Note that as a primary line, HPAEpiC lacks a Cellosaurus accession number. Both normal epithelial lines were grown in Keratinocyte Serum-Free Medium (Gibco #17005042). All culture media were supplemented with 10% heat-inactivated fetal bovine serum (FBS), and cells were maintained in a humidified incubator at 37 °C with 5% CO_2_.

### 2.3. Clinical Study

This study enrolled 117 patients with lung adenocarcinoma female with as history of breast cancer to examine their ER and EGFR status and associated with clinicopathologic factors. All patients with lung adenocarcinoma were registered in the Cancer Registry Group of Tri-Service General Hospital. The study was conducted in accordance with the Declaration of Helsinki, and approved by the Institutional Review Board/Ethics Committee of Tri-Service General Hospital (A202205075 and 6 May 2022 of approval). All patients were followed for mortality data after the date of the diagnosis of breast cancer or lung cancer between January 2015 and May 2023. Patients who were lost to follow-up and those who had insufficient medical record data were excluded. The standard follow-up programs of breast and lung cancers were based on National Comprehensive Cancer Network (NCCN) guidelines. The Kyoto Encyclopedia of Genes and Genomes (KEGG) pathway database was also used to analyze the relationship between breast cancer and lung adenocarcinoma. These mutated EGFR patients all had common EGFR mutations (exon 19 deletion or exon 21 mutation L858R), but not amplifications. And there were no other driver mutations of lung adenocarcinoma, such as ROS-1, BRAF, MET, ALK.

### 2.4. In Vitro Experiments 

Antiestrogen (tamoxifen and fulvestrant), tyrosine kinase inhibitor (TKI) (geftinib), β-estrogendiol were used to treat lung adenocarcinoma and breast cancer cell lines that included EGFR-mutant lung adenocarcinoma cell lines (PC9/HCC827), TKI-resistant lung adenocarcinoma cell lines (PC9GR and HCC827GR), normal lung cell lines (HPAEpiC and BEAS2B), and breast cancer cell line (Au565) and examined the expression of Hb-EGF, EGFR, p-EGFR, ER alpha, ER beta, transforming growth factor (TGF) alpha, proto-oncogene tyrosine-protein kinase Src (SRC), and osteopontin (OPN), p-ERK, p-AKT.

### 2.5. RNA Extraction and Real-Time Polymerase Chain Reaction (PCR)

Total RNAs were extracted by Trizol reagent, and 1 μg of RNA was reversely transcribed using a High-Capacity cDNA Reverse Transcription Kit (Thermo Fisher, MA, USA, # 4368814), according to the manufacturer’s protocols. RNA concentration was measured using NanoDrop 2000. The reverse transcription of total miR-212 was performed using cDNA Synthesis Kit for miRNA (Origene, Kwun Tong, Hong Kong, #HP100042) according to the manufacturer’s protocol. Quantitative analysis of the mature form of miR-212 was performed by SensiFAST™ SYBR No-ROX Kit (Bioline, Indore, India, #98005). The U6 small nuclear RNA was used as an internal control.

### 2.6. Protein Extraction and Western Blot Analysis

Cells were lysed and quantified by a bicinchoninic acid (BCA) protein quantification kit (Thermo Fisher Scientific, Waltham, MA, USA). Proteins were separated by 10% sodium dodecyl-sulfate polyacrylamide gel electrophoresis gel and transferred to polyvinylidene difluoride membranes. Then, the membranes were blocked with 5% non-fat milk for 1 h at room temperature and incubated overnight at 4 °C with the primary antibody. The membranes were incubated with a Horseradish Peroxidase (HRP)-conjugated secondary antibody for 1 h and detected by Enhanced Chemiluminescence (ECL) detection kits system (Pierce, Rockford, IL, USA). In the Hb-EGF release assay, cells (1 × 10^6^ cells/mL) were placed in 6-well plates and allowed to attach overnight. The cells were washed with phosphate-buffered saline and treated with 10 nmol/L estrogen or 10 μmol/L tamoxifen for the indicated time. Supernatants were collected and lyophilized. The lyophilized supernatants were reconstituted to 100 μL and analyzed by Western blotting. 

### 2.7. Cell Migration and Invasion Assays

The wound healing assay was performed to assess cell migration. The migration status was determined by measuring the movement of cells into a scraped area created by a 200-μL pipette tip. The wound closure process was photographed at 24 h. The migration cell lines were tested using a Transwell chamber (8 µm pore; Corning, Inc., Corning, NY, USA). Approximately 1 × 10^6^ cells were added into the upper chamber, and the cell medium was added into the lower chamber. The chambers were then incubated for 24 h at 37 °C with 5% CO_2_. Propidium Iodide (PI) 50 µg/mL was used to stain the migrated cells, which were visualized under an inverted microscope.

### 2.8. Statistical Analysis

GraphPad Prism 7.0 (San Diego, CA, USA) was used in the statistical analyses. Student’s *t* test was utilized to identify differences between two groups. One-way analysis of variance with the Bonferroni post hoc comparison test was used to analyze multiple groups. The Chi-square test is used to determine if there is a statistically significant association between two categorical variables. A significant difference was defined as *p* < 0.05. 

## 3. Results

### 3.1. Hormone Therapy for Previous Breast Cancer Was Significantly Related with the EGFR Status in Lung Adenocarcinoma

Of the 117 patients with lung adenocarcinoma with a history of breast cancer, 38 patients with adequate data were enrolled for further analysis, and other 79 patients with incomplete data were excluded. Table 1 shows that hormone therapy (antiestrogen treatment) for breast cancer is statistically correlated with the expression of mutated EGFR (*p* = 0.021). Other parameters, such as operation, tumor differentiation, location, adjuvant chemotherapy, adjuvant radiation therapy, survival status, relapse status, lymphovascular space invasion status, visceral space invasion status, SUVmax of the tumor, tumor size, carcinoembryonic antigen, number of dissected lymph nodes, and ground-glass opacity (GGO) ratio were not significantly different between patients with wild-type and mutant EGFR. Overall survival was investigated between the hormone therapy for breast cancer and the expression of mutant/wild-type EGFR (Supplementary Figure S1). Although no significant difference (*p* = 0.439) was found between these four groups, the group with wild-type EGFR and without hormone therapy for breast cancer demonstrated better survival.

### 3.2. Tamoxifen Can Upregulate p-EGFR Expression in EGFR-Mutant Lung Adenocarcinoma

**Figure 1:**
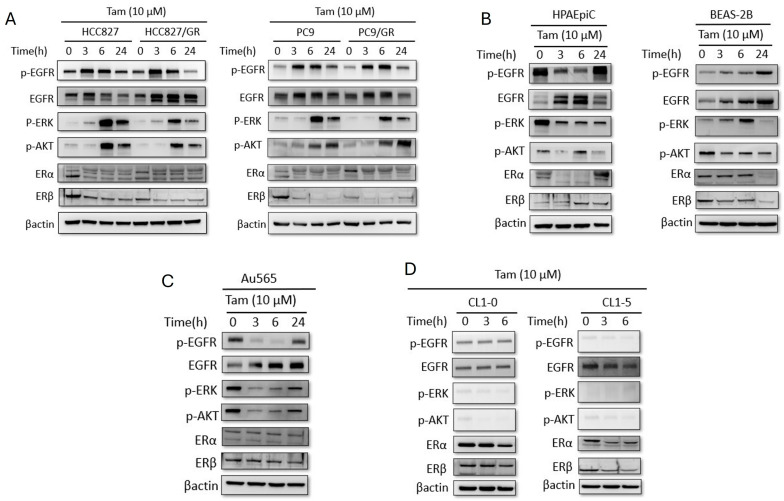
**Tamoxifen can only upregulate p-EGFR in EGFR-mutant lung adenocarcinoma.** (**A**) Western blot analysis of EGFR, ERK, and AKT phosphorylation in sensitive (HCC827, PC9) and gefitinib-resistant (HCC827/GR, PC9/GR) NSCLC cell lines treated with Tamoxifen (10 µM) for 0, 3, 6, and 24 h. Tamoxifen treatment resulted in increased phosphorylation of EGFR, ERK, and AKT in a time-dependent manner. (**B**) Western blot analysis of primary human pulmonary alveolar epithelial cells (HPAEpiC) and immortalized human bronchial epithelial cells (BEAS-2B) treated with Tamoxifen (10 µM). Pathway activation was observed in BEAS-2B but not in primary HPAEpiC cells. (**C**) Western blot analysis of Au565 breast cancer cells treated with Tamoxifen (10 µM), showing time-dependent suppression of p-EGFR, p-ERK, and p-AKT. (**D**) Tamoxifen did not influence p-EGFR in EGFR wild-type lung adenocarcinoma cell lines (CL1-0 and CL1-5). Experiments performed at least in triplicate.

To investigate the off-target effects of Tamoxifen in lung cancer, we treated both EGFR-TKI sensitive cell lines (HCC827, PC9) and their resistant counterparts (HCC827/GR, PC9/GR) with 10 µM Tamoxifen over a 24-h time course. Western blot analysis revealed that Tamoxifen treatment induced a robust, time-dependent phosphorylation of EGFR, AKT, and ERK in both sensitive and resistant NSCLC lines (Figure 1A). This activation typically peaked between 3 and 6 h post-treatment. Interestingly, this phenomenon appeared specific to immortalized or transformed lung cells; while the immortalized lung epithelial line BEAS-2B showed less pathway activation, primary human pulmonary alveolar epithelial cells (HPAEpiC) exhibited no such induction, and potentially a slight suppression of basal signaling (Figure 1B). Furthermore, this effect was distinct from the drug’s canonical mechanism in breast cancer; in Au565 breast cancer cells, Tamoxifen treatment effectively suppressed p-EGFR, p-ERK, and p-AKT levels (Figure 1C), confirming a cell-type-specific divergence in signaling response. Tamoxifen did not influence p-EGFR expression in CL1-0 and CL1-5 cells. Therefore, tamoxifen was found to only upregulate p-EGFR in EGFR-mutant lung adenocarcinoma cell lines (Figure 1D).

### 3.3. Hb-EGF Is Upregulated by Tamoxifen in the ER Signaling Pathway to Activate EGFR Phosphorylation in EGFR-Mutant Lung Adenocarcinoma via Rapid Shedding

**Figure 2:**
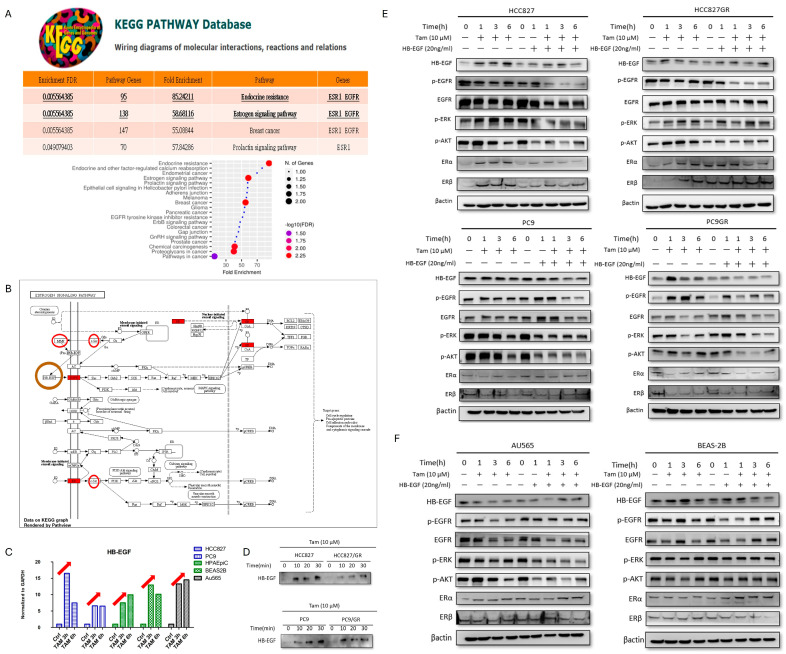
**Hb-EGF is the main affecting factor from the ER pathway to p-EGFR in lung adenocarcinoma.** (**A**) In the KEGG pathway database, endocrine resistance and the estrogen signaling pathway were most associated with enrichment with ER and EGFR. (**B**) The estrogen signaling pathway by KEGG analysis (fold enrichment 58.68) was most associated with ER and EGFR enrichment. The possible interacting factors included Hb-EGF, TGF, and SRC, which were circled. And ER was covered with red background. (**C**) In the examination of the RNA expression of Hb-EGF in tamoxifen-treated cell lines (HCC827, PC9, HPAEpic, BEAS-2B, and Au565), only Hb-EGF was stimulated, and these cell lines showed consistent upregulation by the red arrow. (**D**) The supernatant of tamoxifen-treated lung adenocarcinoma cell lines was collected because Hb-EGF was exhibited in the extracellular matrix. Hb-EGF was still upregulated after tamoxifen treatment. (**E**) Comparison of signaling pathway activation in lung cancer cells (HCC827, PC9, and their GR variants) treated with either Tamoxifen (10 µM) or recombinant HB-EGF (20 ng/mL) for the indicated times. Tamoxifen induced phosphorylation patterns of EGFR, ERK, and AKT similar to those induced by exogenous HB-EGF. (**F**) Comparative analysis of signaling in Au565 (breast cancer) and BEAS-2B (lung epithelial) cells. While HB-EGF activated the pathway in both lines, Tamoxifen only mimicked this activation in BEAS-2B cells, whereas it inhibited the pathway in Au565 cells. Experiments performed at least in triplicate.

To determine the interaction of ER and EGFR, the KEGG pathway database (Figure 2A) was used to investigate these key factors. Endocrine resistance (fold enrichment 85.24) and estrogen signaling pathway (fold enrichment 58.68) were most associated with ER and EGFR enrichment. The possible interacting factors included Hb-EGF, TGF, and SRC (Figure 2B). In the literature review, the possible predictors were searched between ER and EGFR in lung adenocarcinoma. OPN [15,16] was found to play an important role in the occurrence and development of breast cancer and non-small cell carcinoma through different mechanisms. Therefore, OPN was also considered a possible key factor between the signaling pathway of ER and EGFR in lung adenocarcinoma.

In Figure 2C and Supplementary Figure S2A–C, RNA expressions of Hb-EGF, TGF, SRC, and OPN were examined in tamoxifen-treated cell lines. Only Hb-EGF was consistently upregulated in lung adenocarcinoma, normal, and breast cancer cell lines. Thus, we try to prove that Hb-EGF was the main affecting factor from the ER pathway to p-EGFR in lung adenocarcinoma. In Figure 2D, the supernatant of tamoxifen-treated lung adenocarcinoma cell lines was collected because active soluble Hb-EGF was exhibited in the extracellular matrix. We hypothesized that Tamoxifen activates EGFR signaling through the release of ligands. We observed a rapid increase in HB-EGF protein levels within 10 to 30 min of Tamoxifen exposure in both HCC827 and PC9 parental and resistant lines.

HB-EGF neutralizing polyclonal antibodies were used to utilize its antiproliferative properties in vitro [17]. Comparison of signaling pathway activation in lung cancer cells (HCC827, PC9, and their GR variants) treated with either Tamoxifen (10 µM) or recombinant HB-EGF (20 ng/mL) for the indicated times. Tamoxifen induced phosphorylation patterns of EGFR, ERK, and AKT similar to those induced by exogenous HB-EGF (Figure 2E). Comparative analysis of signaling in Au565 (breast cancer) and BEAS-2B (lung epithelial) cells. While HB-EGF activated the pathway in both lines, Tamoxifen only mimicked this activation in BEAS-2B cells, whereas it inhibited the pathway in Au565 cells (Figure 2F).

In the ER signaling pathway, tamoxifen activates the phosphorylation of EGFR, and increased Hb-EGF secretion can overcome the neutralizing effect of Hb-EGF antibody in EGFR-mutant lung adenocarcinoma. Thus, we proved the effect of tamoxifen and the key role of Hb-EGF between the ER and EGFR signaling pathways in mutant EGFR lung adenocarcinoma. Furthermore, we wanted to investigate cell proliferation and tumor invasion in tamoxifen-treated these cell lines.

**Figure 3:**
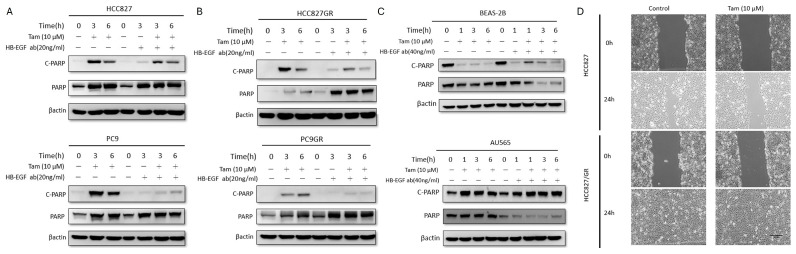
**Tamoxifen inhibited cell migration and increased the expression of the apoptotic marker c-PARP. HB-EGF neutralization can reduce cell apoptosis.** (**A**) Western blot analysis of apoptosis marker Cleaved PARP (C-PARP) in HCC827 and PC9 cells treated with Tamoxifen (10 µM) alone or in combination with an HB-EGF neutralizing antibody (20 ng/mL) for 0, 3, and 6 h. Co-treatment with the neutralizing antibody attenuated Tamoxifen-induced PARP cleavage. (**B**) Western blot analysis of C-PARP in resistant HCC827/GR and PC9/GR cells under identical conditions, showing a similar rescue effect by the HB-EGF antibody. (**C**) Western blot analysis of C-PARP in BEAS-2B and AU565 cells treated with Tamoxifen (10 µM) with or without HB-EGF neutralizing antibody (40 ng/mL). Hb-EGF neutralization can increase c-PARP in normal lung cell lines (BEAS-2B). The apoptotic factor c-PARP is mildly increased under tamoxifen treatment. Hb-EGF neutralization has no role in cell apoptosis in HER2/neu-positive breast adenocarcinoma cell line (AU565). (**D**) The wound healing tests of tamoxifen-treated HCC827/HCC827GR cells showed the inhibition of cell migration, particularly in HCC827 cells. Experiments performed at least in triplicate.

To determine the functional consequence of this pathway activation, we assessed apoptosis via PARP cleavage (C-PARP). Tamoxifen treatment (10 µM) strongly induced PARP cleavage at 3 and 6 h in HCC827, PC9, and their resistant derivatives (Figure 3A,B). Strikingly, blocking extracellular HB-EGF with a neutralizing antibody significantly attenuated PARP cleavage in all cancer cell lines. This rescue effect was also observed in BEAS-2B cells (Figure 3C). The neutralization of Hb-EGF slightly increased the expression of c-PARP in BEAS-2B. For AU565, the expression of the apoptotic factor c-PARP mildly increased after Tamoxifen treatment. The neutralization of Hb-EGF has no role on cell apoptosis in AU565 (Figure 3C). These data suggest that the Tamoxifen-induced shedding of HB-EGF and subsequent EGFR pathway activation is not a pro-survival response, but rather a critical mediator of Tamoxifen-induced cytotoxicity in these lung cells. The wound healing tests of HCC827/HCC827GR (Figure 3D) and PC9/PC9GR (Supplementary Figure S3) treated with tamoxifen showed more inhibition of cell migration than the control group and this correlated with the results shown in Figure 3A,B demonstrating more apoptotic activity. Therefore, we proved that tamoxifen can also inhibit cell migration and promote cell apoptosis in EGFR-mutant lung adenocarcinoma. Hb-EGF was considered to play a key role in cell apoptosis.

### 3.4. Tamoxifen Can Overcome Estrogen Effect and Restore p-EGFR Upregulation Only in EGFR-Mutant Lung Adenocarcinoma

To mimic the clinical condition of lung adenocarcinoma and breast cancer, tamoxifen was added to long-term estrogen treatment in EGFR-mutant lung adenocarcinoma cell lines. We try to clarify the effect of estrogen on p-EGFR in these cell lines. Treatment with Estradiol (beta-E2, 10 nM) alone does not induce the same robust activation of p-EGFR or upregulation of HB-EGF compared to Tamoxifen. In fact, in HCC827 and PC9 cells (sensitive and resistant), beta-E2 seems to have a slightly suppressive effect on basal p-EGFR compared to controls, and it does not trigger the massive upregulation of HB-EGF seen with Tamoxifen. It also does not induce Bax (pro-apoptotic marker) significantly. In Supplementary Figure S4A, estrogen did not influence the level of p-EGFR at CL1-5, BEAS-2B, and HPAEpiC; however, it decreased the level of p-EGFR in the breast adenocarcinoma cell line (Au565).

**Figure 4:**
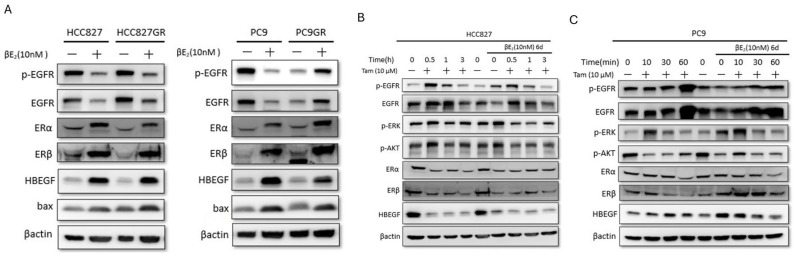
**Tamoxifen can overcome the estrogen effect and restore the upregulation of p-EGFR in EGFR-mutant lung adenocarcinoma.** (**A**) Western blot analysis of p-EGFR, HB-EGF, and Bax expression in HCC827, PC9, and their GR variants treated with Estradiol (beta-E2, 10 nM) for 24 h. beta-E2 treatment alone did not induce HB-EGF upregulation or Bax expression. (4B) and (4C) Western blot analysis of signaling pathways in HCC827 (**B**) and PC9 (**C**) cells treated with Tamoxifen (10 µM) alone or following long-term (6 days) pre-treatment with beta-E2 (10 nM). Tamoxifen-induced upregulation of HB-EGF and phosphorylation of EGFR, ERK, and AKT were slightly suppressed after long-term estrogen exposure. Experiments performed at least in triplicate.

Then, HCC827 (Figure 4B) and PC9 (Figure 4C) cells were treated with estrogen for 6 days. These blots appear to test if long-term estrogen treatment modulates the Tamoxifen effect. Rapid induction of p-EGFR, p-ERK, p-AKT, and HB-EGF (crucial finding: HB-EGF band intensity increases strongly) is confirmed by only Tamoxifen treatment (left panel). Long-term exposure (6 days) to beta-E2 does slightly block the Tamoxifen-induced upregulation of p-EGFR or HB-EGF (right panel). The activation became less strong. This suggests the Tamoxifen effect is likely dependent on classical ER genomic signaling. Similar effects were found on CL1-5 and AU565 (Supplementary Figure S4B,C). However, no effects were noted on the p-EGFR level in normal lung cell lines (Supplementary Figure S4D,E). Here, Hb-EGF was not tested using the extracellular matrix, so the results might not reflect the reality.

In addition, we wanted to know the function of another antiestrogen drug (fulvestrant) because it has nearly total antagonist effect. As shown in Supplementary Figure S5A–D, fulvestrant demonstrated similar effects to increase p-EGFR expression only in EGFR-mutant lung adenocarcinoma cell lines.

### 3.5. The Combined Administration of Tamoxifen and Geftinib in EGFR-Mutant Lung Adenocarcinoma Showed More Potential Inhibition of p-EGFR

**Figure 5:**
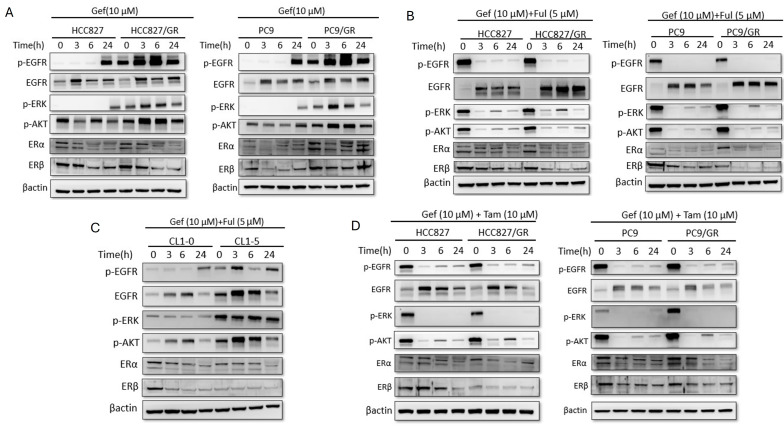
**The coadministration of tamoxifen and geftinib in EGFR-mutant lung adenocarcinoma showed more potential inhibition of p-EGFR.** (**A**) Western blot analysis of EGFR signaling in HCC827, PC9, and their resistant (GR) counterparts treated with Gefitinib (10 µM) alone. Resistant cells exhibited sustained or rebounding p-EGFR and p-ERK levels at 24 h. Geftinib suppressed ERβ expression. (**B**) Geftinib (10 μM) and pure anti-estrogen fulvestrant (5 μM) were administered to treat EGFR-mutant lung adenocarcinoma (HCC827/HCC827GR and PC9/PC9GR). The combination of Gefitinib and Fulvestrant showed enhanced suppression of signaling in parental and GR cells. (**C**) Western blot analysis of CL1-0 (low invasion) and CL1-5 (high invasion) lung cancer cells treated with Gefitinib (10 µM) and Fulvestrant (5 µM). The expression of p-EGFR was not significantly suppressed. (**D**) Western blot analysis of HCC827/GR and PC9/GR cells treated with the combination of Gefitinib (10 µM) and Tamoxifen (10 µM). The combination treatment resulted in a more durable suppression of p-EGFR, p-ERK, and p-AKT compared to Gefitinib alone, effectively overcoming resistance signals at 24 h. Experiments performed at least in triplicate.

Given that Tamoxifen activates EGFR via HB-EGF shedding, we investigated how this interacts with EGFR TKI (geftinib). In Figure 5A, treatment with the EGFR-TKI Gefitinib (10 µM) effectively silenced signaling in sensitive cells (HCC827, PC9). However, in the resistant variants (HCC827/GR, PC9/GR), while there was initial suppression at 3–6 h, a strong “rebound” of p-EGFR, p-ERK, and p-AKT was observed at 24 h.

Then, geftinib and fulvestrant combination was used to treat EGFR-mutant lung adenocarcinoma cell lines (Figure 5B). The pure anti-estrogen Fulvestrant (5 µM) enhanced Gefitinib efficacy, suppressing the 24-h slightly rebound in resistant cells, though Tamoxifen appeared more potent in complete signal abrogation (p-ERK and p-AKT). In Figure 5C, CL1-5 (highly invasive) and CL1-0 (less invasive), the combination of Gef + Ful leads to no suppression of p-EGFR and p-ERK over time, suggesting no or less involvement of ER signaling in the wild-type EGFR mutation cell lines. However, the combination of geftinib and fulvestrant still strongly suppressed p-EGFR level in normal lung and breast cancer (Supplementary Figure S6A,B) cell lines.

As shown in Figure 5D, Tamoxifen (10 µM) was combined with Gefitinib, the “rebound” seen in resistant cells was completely abolished. at 24 h, p-EGFR, p-ERK, and p-AKT levels were undetectable or barely visible in the GR cell lines. Therefore, the combination of geftinib with tamoxifen or fulvestrant can overcome EGFR TKI resistance in mutated EGFR lung adenocarcinoma.

## 4. Discussion

### 4.1. Relationships Between Breast Cancer and Lung Cancer from Clinical Studies

According to the Taiwan cancer registry annual report in 2020 [1], lung cancer was the leading cause of death of all cancers, with mortality rate of 42.8 per 100,000 people. Breast cancer was also the leading cause of death in women. Over time, the diagnosis and treatment further improved in lung and breast cancers. However, the mortality rate increased annually. In clinical practice, some common features were found between breast cancer and lung adenocarcinoma, such as female predominance, ER expression, and EGFR expression, during carcinogenesis [2,3,4,5,6]. In recent years, some studies [7,8,9,10] have reported that patients with lung adenocarcinoma who had a history of breast cancer presented close relationships between ER expression and EGFR. Although the relation between estrogen and lung cancer risk is controversial [18,19,20], a recent study from the Taiwan National Health Insurance Research Database [21] indicated that patients with breast cancer had a significantly higher risk of developing secondary primary lung cancer than those without breast cancer, particularly in younger groups and those without any comorbidities. In addition, Dr. Hu et al. presented a retrospective cohort study [10] of 159 patients with lung and breast cancer and showed that ER and progesterone receptor (PR) expression significantly correlated with EGFR gene mutation in lung cancer with simultaneous primary breast cancer. In our lung cancer database, an increasing number of patients with breast cancer had second primary lung cancer. Therefore, our female patients with lung adenocarcinoma with history of breast cancer were collected, and the risk factors of local recurrences and distant metastases were investigated [9]. We investigated 38 patients with lung adenocarcinoma preceding breast cancer, excluding loss of follow-up and inadequate data, for the relationship between EGFR mutation status in lung adenocarcinoma and hormone therapy for breast cancer. The results revealed 72.22% EGFR mutation in lung adenocarcinoma in patients with breast cancer with hormone therapy (*p* = 0.021) (Table 1). In addition, overall survival was investigated between the hormone therapy for breast cancer and the expression of mutant/wild-type EGFR. Although no significant difference (*p* = 0.206) was found among the four groups, the group with wild-type EGFR and no hormone therapy for breast cancer had better survival (Figure 1). Thus, a special association between breast cancer and second primary lung cancer has been discovered in clinical practice, particularly in Asian women. The mechanism of interaction between ER and EGFR in lung adenocarcinoma was still not well acknowledged. Furthermore, a potential limitation of this study is that the status of RAS mutations (including KRAS, NRAS, and HRAS) was not comprehensively analyzed in our patient cohort. In clinical practice, RAS mutations are frequently utilized to select appropriate treatment strategies, as they are known to be significant drivers of oncogenesis and can confer primary resistance to EGFR-targeted therapies. Since RAS signaling operates downstream of EGFR, the presence of concurrent RAS mutations could potentially bypass the EGFR pathway and confound the observed crosstalk between the ER and EGFR signaling axes. Consequently, the lack of RAS mutation data might influence our conclusions regarding the efficacy of combined antiestrogen and TKI therapy, suggesting that future studies should include integrated molecular profiling to fully elucidate these interactions.

### 4.2. Crosstalk Between ER and EGFR in Lung Cancer

According to a literature review [22], ERβ is the main mediating receptor for the physiological effects of estrogen on the lung. Dr. Yu et al. [23] presented that ERβ positivity by immunohistochemistry (IHC) staining was significantly related to poor survival in NSCLC. Although Prof. Stabile et al. [24] identified high ERβ1 and PR expression in patients with lung cancer with poor outcomes, their literature review showed equivocal results that ERβ expression was associated with lung cancer survival. Therefore, ER was also seen as a therapeutic target in lung cancer. Dr. Rodriguez-Lara et al. [25] reported synergistic effects of antiestrogen treatment and EGFR TKI in their review of preclinical studies and clinical trials.

In vitro studies [4,26,27] have also shown that ERβ1 expression was responsible for EGFR TKI resistance in EGFR-mutant lung adenocarcinoma. Prof. Siegfried et al. [28] combined the multitargeted TKI vandetanib with the antiestrogen fulvestrant to enhance their antitumor effects on NSCLC. These findings indicated a potential interaction between ER and EGFR in lung adenocarcinoma.

The crosstalk between EGFR and ER in breast cancer cell lines was identified [11,14]. The model proposed E2-dependent ER-mediated transactivation of EGFR through a G-protein-dependent mechanism, and estradiol-bound ER stimulated Src activity, which in turn activated metalloproteinases 2 and 9, increasing Hb-EGF release. Hb-EGF is the main ligand that binds to EGFR and activates it signaling. The interaction of ER and EGFR in cerebral aneurysm pathogenesis was also proposed in the similar pathway [13]. However, some studies [11,12] have presented that estrogen can increase Hb-EGF secretion in breast and lung adenocarcinoma cell lines. However, the expression of EGFR level was suppressed by estrogen and increased by fulvestrant [12]. This result appears contradictory because increasing Hb-EGF expression by estrogen should stimulate EGFR. Thus, we wanted to investigate the function of Hb-EGF between ER and EGFR in lung adenocarcinoma.

### 4.3. Effect of Hb-EGF Between ER and EGFR

Hb-EGF is the ligand of EGF receptors and influences cell cycle progression, molecular chaperone regulation, cell survival, cellular functions, adhesion, and mediation of cell migration. Therefore, Hb-EGF antibodies have antiproliferative effects [17]. However, the role of Hb-EGF in EGFR-mutant lung adenocarcinoma is different. Tamoxifen induced Hb-EGF secretion and stimulated p-EGFR expression with increasing cell apoptosis and inhibition of cell migration (Figure 3A,B,D and Supplementary Figure S3). Thus, we are doubtful of the conflicting result of increasing p-EGFR expression and cell apoptosis by tamoxifen treatment. This question should be clarified in further experiments. We hypothesized that tamoxifen cytotoxicity is stronger than increasing p-EGFR effect in EGFR-mutant lung adenocarcinoma. In Figure 3A,B, Hb-EGF was found to be responsible for cell apoptosis in EGFR-mutant lung adenocarcinoma by combination of tamoxifen and anti-Hb-EGF antibodies.

As Hb-EGF is involved in the crosstalk of ER and EGFR in breast cancer [14], cardiovascular diseases [29,30], and germ cell [31], the soluble mature HB-EGF is cleaved from the membrane-associated pro-Hb-EGF by matrix metalloproteinase through ectodomain shedding. However, there is no well-defined crosstalk about Hb-EGF in lung adenocarcinoma in literature reviews [14,29,30,31]. Thus, we proved that Hb-EGF is the key predictor between ER and EGFR in lung adenocarcinoma. Furthermore, we also want to identify the associated apoptosis in the Hb-EGF pathway in EGFR-mutant lung adenocarcinoma. According to literature reviews, Hb-EGF has double roles in apoptosis and proliferation. In the present study, Hb-EGF was stimulated by antiestrogen drugs in EGFR-mutant lung adenocarcinoma cell lines with related cell apoptosis. 

Cells are treated with single-agent gefitinib, single-agent tamoxifen, and the combination. Western blot is performed to assess the expression levels of p-EGFR. The combination must demonstrate a significantly greater reduction in the expression of p-EGFR compared to a sum of effects exerted by agents when used alone, providing direct molecular proof that the drugs in suppressing the key oncogenic driver. Tamoxifen can overcome the estrogen effect and restore the upregulation of p-EGFR in lung adenocarcinoma, and combined administration of tamoxifen and geftinib in EGFR-mutant lung adenocarcinoma potentially inhibited p-EGFR expression. The dual blockade of both the EGFR and Estrogen Receptor (ER) pathways (or the ER-mediated crosstalk) by the combination is what drives the enhanced p-EGFR inhibition and more cytotoxic effect. The more potentially blocking the EGFR pathway is needed to confirm by looking at its critical downstream survival and proliferation signals (PI3K/AKT Pathway and RAS/MAPK Pathway).

In the clinical results, many confounding factors were identified, such as cancer interval, duration of medications, and menopause effect. This study also has some limitations, such as the small sample size and retrospective design, which bring about random variations and low statistical power. Further studies enrolling a large number of patients with lung adenocarcinoma and breast cancer who matched the inclusion criteria and better distribution across cancer subtypes are needed to identify the clinical pathological factors and expression of target proteins in EGFR-mutant or wild-type lung adenocarcinoma in clinical practice and improve lung cancer treatment with antiestrogen for specific conditions (maybe ER strong positive).

## 5. Conclusions

Hormone therapy for breast cancer in patients with a history of LUAD was significantly correlated with the expression of mutated EGFR in the lung cancer. Tamoxifen alone was found to upregulate p-EGFR in EGFR-mutant LUAD cell lines (HCC827/PC9). This effect is mediated by the upregulation of Heparin-binding EGF-like growth factor (Hb-EGF), a key ligand that stimulates EGFR phosphorylation. Tamoxifen also increased cell apoptosis and inhibited cell migration in these cells. Tamoxifen was also able to overcome the inhibitory effect of estrogen and restore the upregulation of p-EGFR in EGFR-mutant LUAD. Coadministration of tamoxifen and the EGFR TKI gefitinib more potentially inhibited p-EGFR expression in EGFR-mutant LUAD cell lines (HCC827/PC9). This dual blockade of the ER and EGFR pathways is what drives the enhanced p-EGFR inhibition. The combination also showed greater inhibition in the TKI-resistant cell lines (HCC827GR and PC9GR), suggesting tamoxifen may overcome TKI resistance. The strong molecular and in vitro evidence suggests that for patients diagnosed with both ER-positive breast cancer and EGFR-mutant LUAD, the combination of tamoxifen and EGFR TKI may offer an effective targeted treatment strategy. Further clinical studies are needed to confirm the clinical pathological factors and treatment response for this specific patient population.

## Figures and Tables

**Table 1:** Characteristics of lung adenocarcinoma patients with previous breast cancer.

Variable	Wild Type EGFR n = 18 (%)	Mutated EGFR n = 20 (%)	*p*-Value ^a^
Operation Anatomic resection Sublobar resection	11 (61.11) 7 (38.89)	13 (65) 7 (35)	0.74
Differentiation Well/Moderate Poor	10 (55.56) 8 (44.44)	10 (50) 10 (50)	0.74
Location Central Peripheral	7 (38.89) 11 (61.11)	8 (40) 12 (60)	0.946
Adjuvant chemotherapy Yes No	6 (33.33) 12 (66.67)	5 (25) 15 (75)	0.584
Adjuvant radiotherapy Yes No	1 (5.56) 17 (94.44)	0 20 (100)	0.298
Survival Yes No	17 (94.44) 1 (5.56)	18 (90) 2 (10)	0.423
Relapse Yes No	3 (16.67) 15 (83.33)	4 (20) 16 (80)	0.798
LVSI Absent Present	16 (88.89) 2 (11.11)	19 (95) 1 (5)	0.499
VPI Absent Present	15 (83.33) 3 (16.67)	20 (100) 0	0.059
HT for breast CA Yes No	5 (27.78) 13 (72.22)	13 (65) 7 (35)	0.021 ^a^
RT for breast CA Yes No	9 (50) 9 (50)	11 (55) 9 (45)	0.766
Chemotherapy for breast CA Yes No	8 (44.44) 10 (55.56)	10 (50) 10 (50)	0.74
Age (years-old)	56.28 ± 5.98	62.6 ± 11.75	0.047 ^b^
SUVmax of tumor	3.19 ± 3.85	2.71 ± 2.03	0.662
Tumor size (cm)	1.24 ± 0.69	1.67 ± 0.98	0.151
CEA (ng/mL)	6.31 ± 14.23	2.51 ± 2.38	0.247
Dissected lymph nodes	7.29 ± 4.63	9.47 ± 4.89	0.18
GGO ratio	0.42 ± 0.42	0.52 ± 0.39	0.431

^a^ Significance was assessed using χ^2^ tests. Key: LVSI, lymphovascular space invasion; p-stage, pathology stage; VPI, visceral pleural invasion; HT, hormone therapy; RT, radiation therapy; CA, cancer. ^b^ Significance was assessed using Student’s *t* tests. Key: SUVmax, maximum standard uptake value of FDG; CEA, carcinoembryonic antigen; GGO, ground-glass opacity.

## Data Availability

Data available on request from the first author or corresponding author.

## Supplementary Materials

The following supporting information can be downloaded at: https://www.mdpi.com/article/10.3390/cells15020098/s1, Figure S1: The Kaplan-Mierer curve of overall survival of patients with lung adenocarcinoma and breast cancer. Figure S2: The RNA expression of TGFα, SRC, OPN in Tamoxifen treated cell lines (HCC827, PC9, BEAS-2B). Figure S3: The wound healing test of PC9/PC9GR after Tamoxifen treatment. Figure S4: The combination effects of estrogen and Tamoxifen in EGFR wild type, breast cancer, normal lung cell lines. Figure S5: The p-EGFR level after Fulvestrant treatment in cell lines. Figure S6: The combination effect of Geftinib and Fulvestrant in breast cancer and normal lung cell lines.

## Abbreviations

**EGFR**: Epidermal growth factor receptor. **ER**: Estrogen receptor. **TKI**: Tyrosine kinase inhibitor. **p-EGFR**: Phosphorylated epidermal growth factor receptor. **Hb-EGF**: Heparin-binding epidermal growth factor-like growth factor. **TGF**: Transforming growth factor. **SRC**: Proto-oncogene tyrosine-protein kinase Src. **p-ERK**: Phospho-extracellular signal-regulated kinase. **p-AKT**: Phospho-protein kinase B. **FBS**: Fetal bovine serum. **KEGG**: Kyoto Encyclopedia of Genes and Genomes. **PCR**: Polymerase chain reaction. **PBS**: Phosphate-buffered saline. **LVSI**: Lymphovascular space invasion. **VPI**: Visceral pleural invasion. **SUVmax**: Maximum standard uptake value. **CEA**: Carcinoembryonic antigen. **C-PARP/c-PARP**: Cleaved poly (ADP-ribose) polymerase. **beta-E2**: Estradiol.
