# Supplementary figures and images for: The Crosstalk Mechanism of EGFR and ER in EGFR-Mutant Lung Adenocarcinoma
Source: Cells. 2026 Jan 6;15(2):98. doi: 10.3390/cells15020098 (PMC12838910; doi:10.3390/cells15020098)

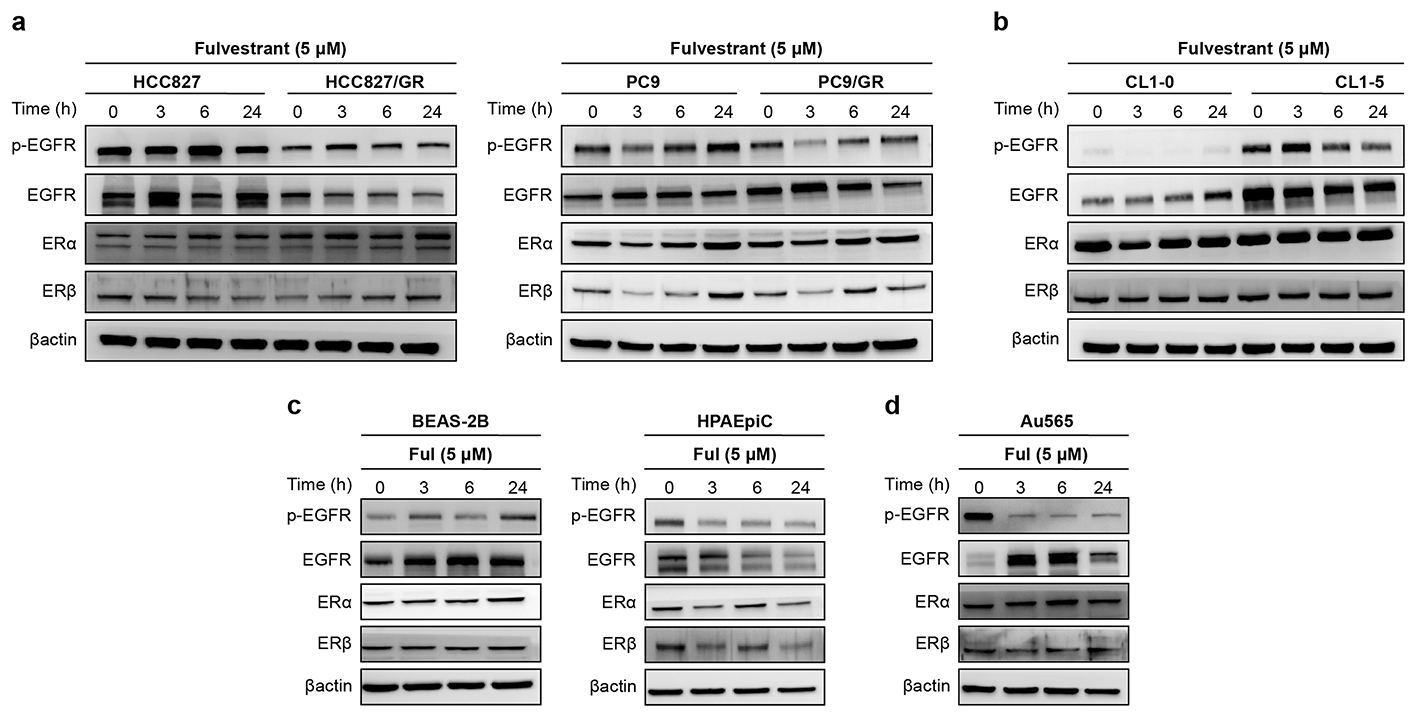

Supplement: Supplementary file 1 [file cells-15-00098-s001.zip › Figure S5.tif]

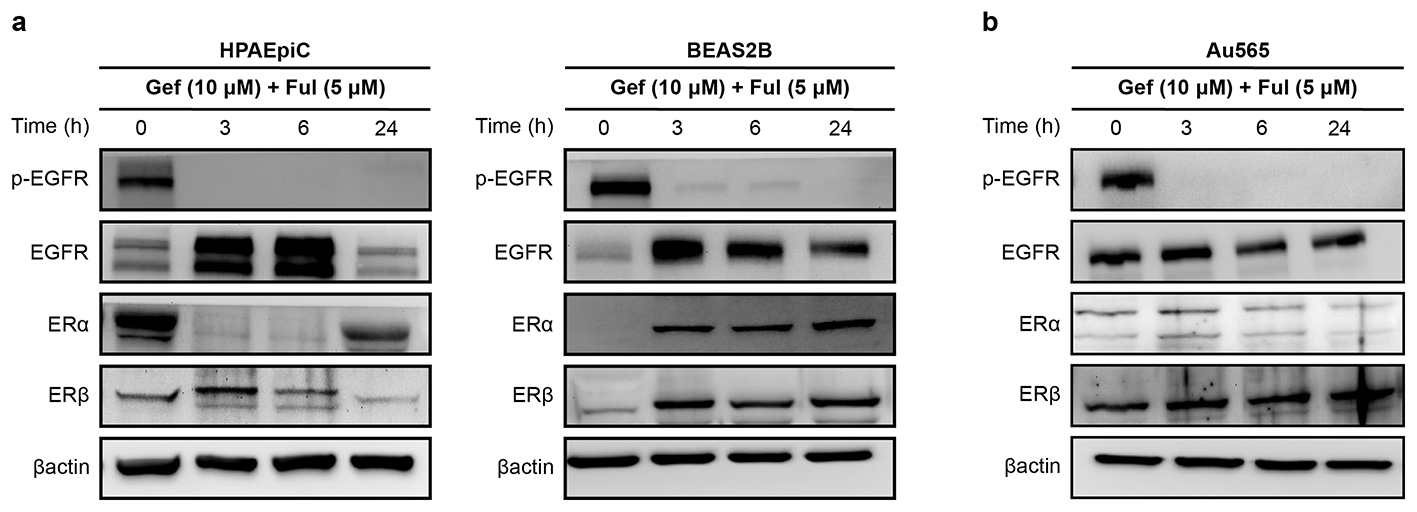

Supplement: Supplementary file 1 [file cells-15-00098-s001.zip › Figure S6.tif]

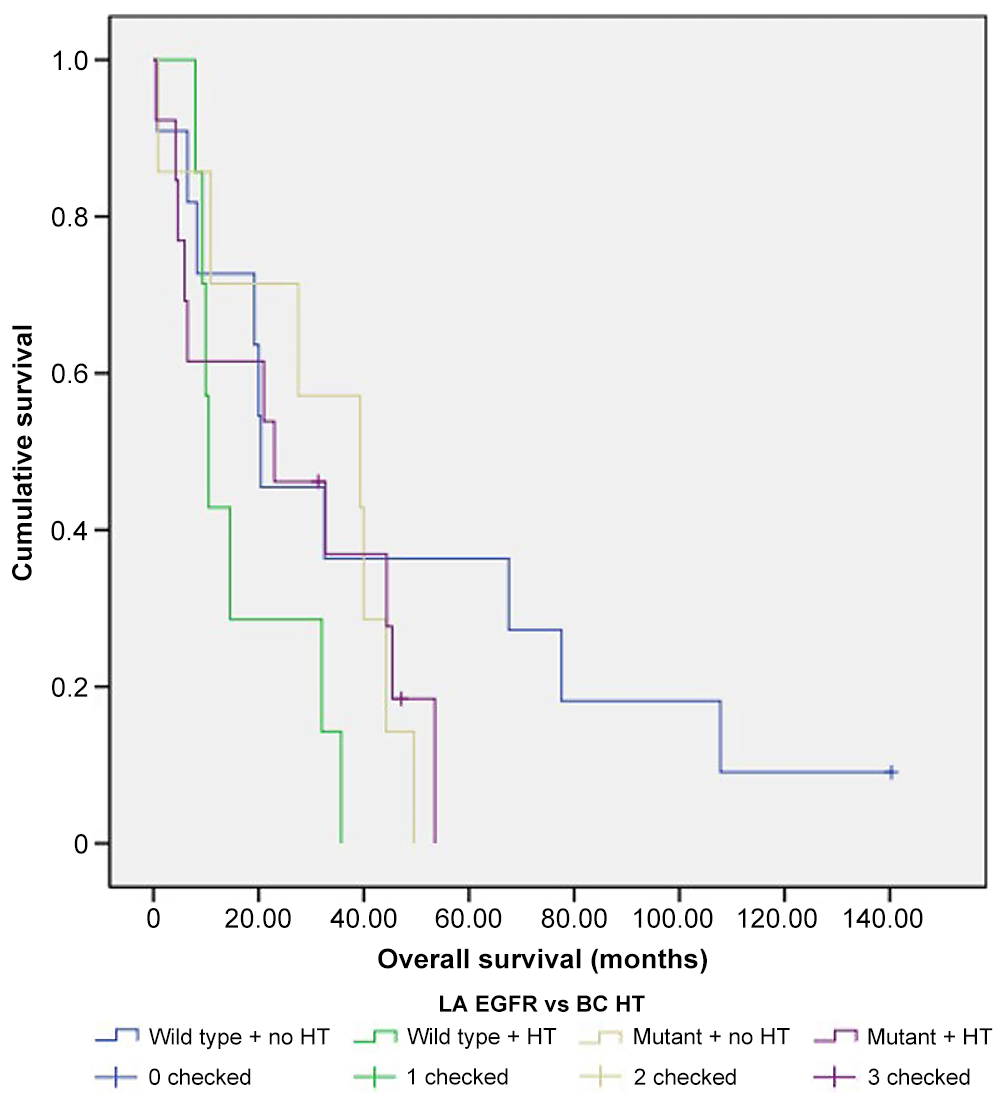

Supplement: Supplementary file 1 [file cells-15-00098-s001.zip › Figure S1.tif]

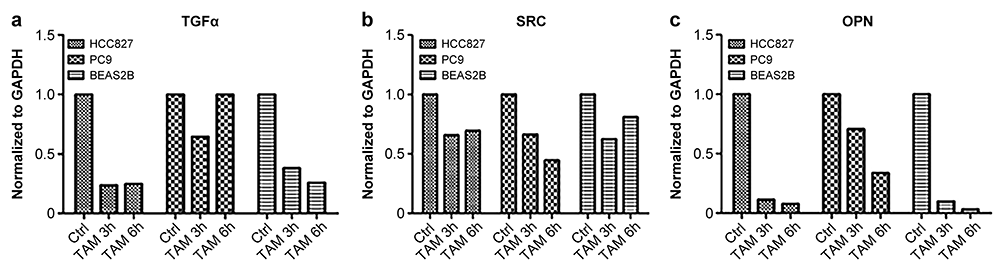

Supplement: Supplementary file 1 [file cells-15-00098-s001.zip › Figure S2.tif]

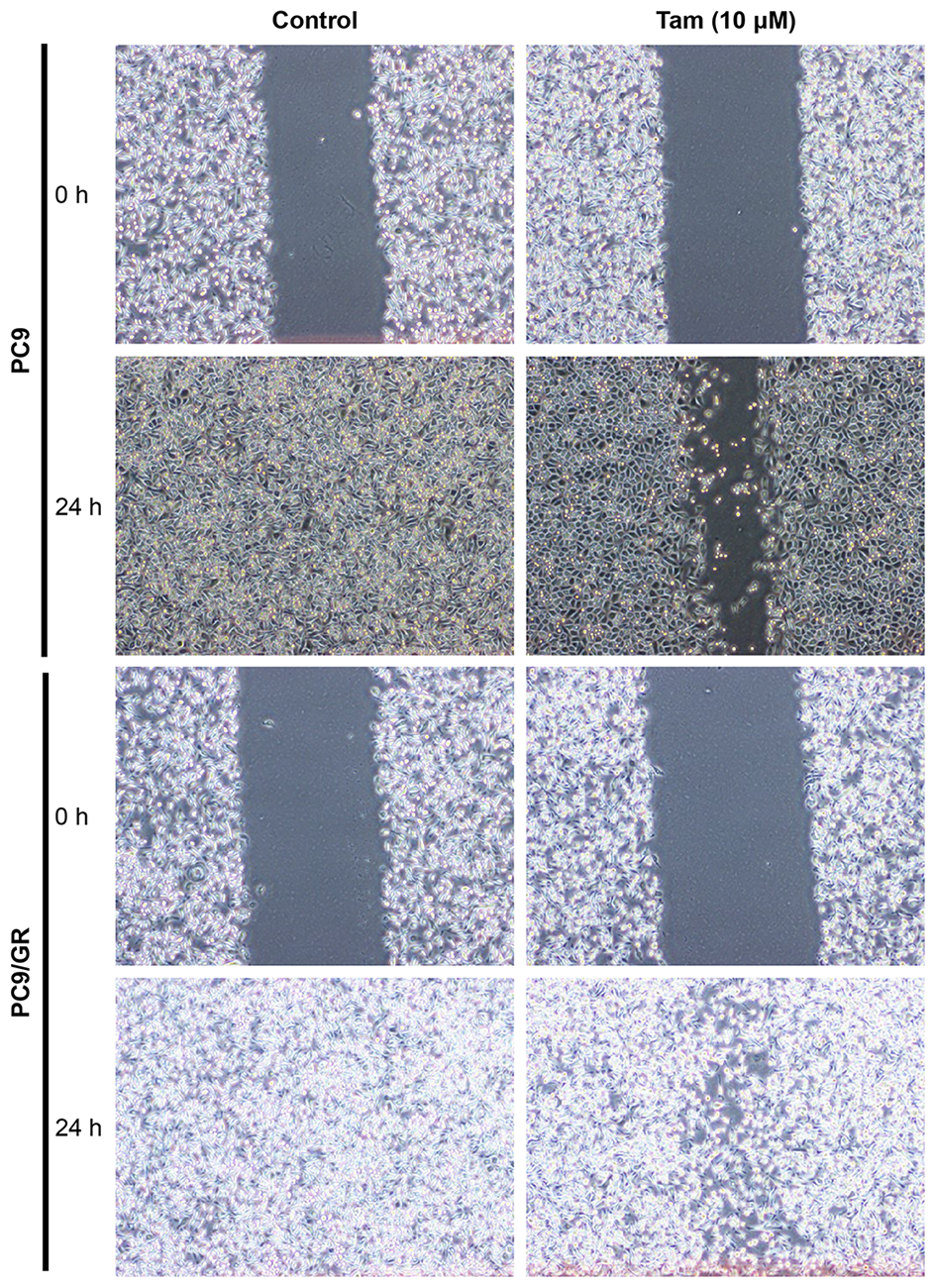

Supplement: Supplementary file 1 [file cells-15-00098-s001.zip › Figure S3.tif]

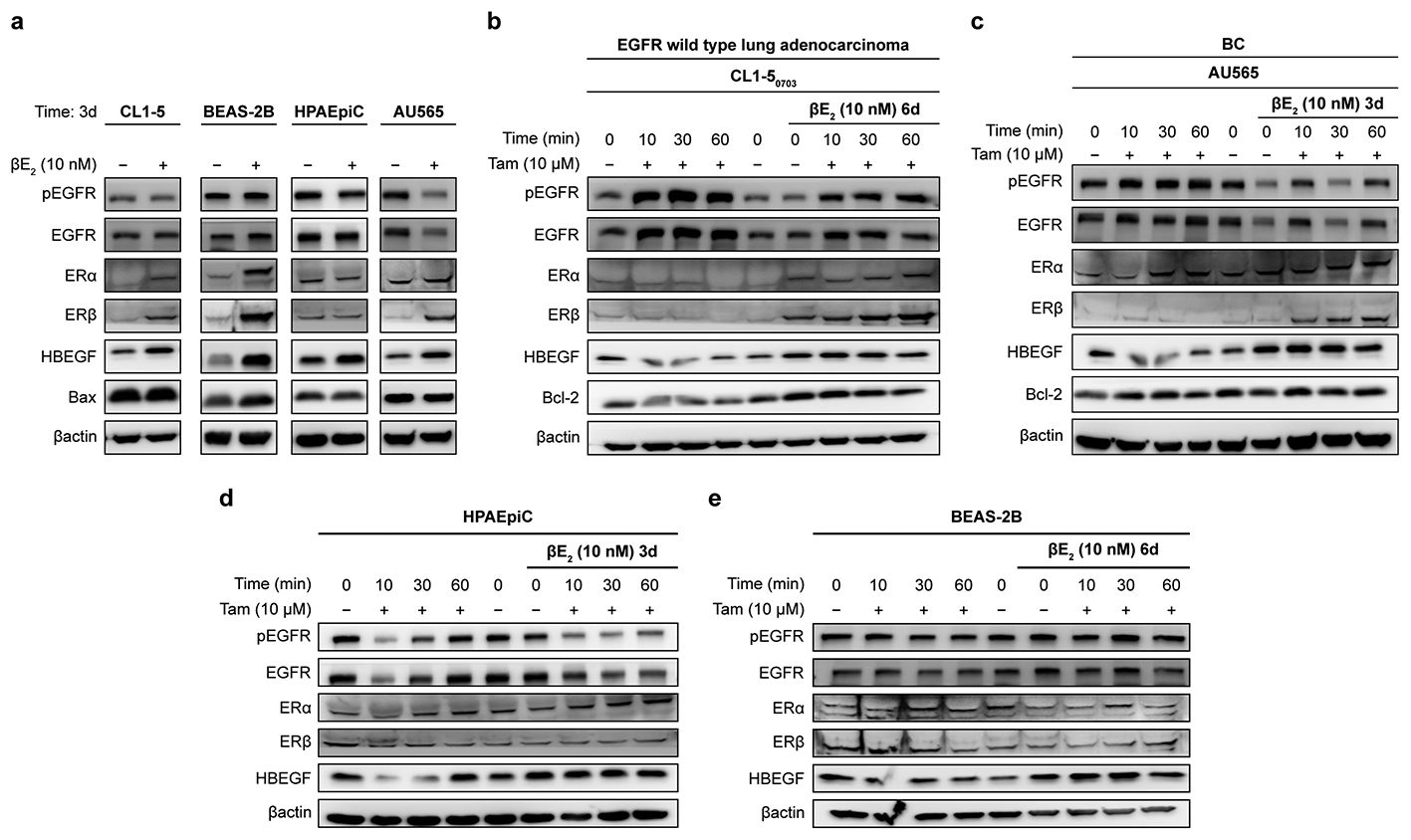

Supplement: Supplementary file 1 [file cells-15-00098-s001.zip › Figure S4.tif]
